# The power of green: Harnessing phytoremediation to combat micro/nanoplastics

**DOI:** 10.1016/j.eehl.2024.04.001

**Published:** 2024-04-16

**Authors:** Wenke Yuan, Elvis Genbo Xu, Soha Shabaka, Peng Chen, Yuyi Yang

**Affiliations:** a Hubei Key Laboratory of Wetland Evolution & Ecological Restoration, Wuhan Botanical Garden, Chinese Academy of Sciences, Wuhan 430074, China; b Key Laboratory of Lake and Watershed Science for Water Security, Nanjing Institute of Geography and Limnology, Chinese Academy of Sciences, Nanjing 210008, China; c Danjiangkou Wetland Ecosystem Field Scientific Observation and Research Station, Chinese Academy of Sciences & Hubei Province, Wuhan 430074, China; d Department of Biology, University of Southern Denmark, Odense 5230, Denmark; e National Institute of Oceanography and Fisheries, Cairo 11516, Egypt

**Keywords:** Environmental sustainability, Hyperaccumulator, Microplastics, Remediation strategies, Technical advances

## Abstract

Plastic pollution and its potential risks have been raising public concerns as a global environmental issue. Global plastic waste may double by 2030, posing a significant challenge to the remediation of environmental plastics. In addition to finding alternative products and managing plastic emission sources, effective removal technologies are crucial to mitigate the negative impact of plastic pollution. However, current remediation strategies, including physical, chemical, and biological measures, are unable to compete with the surging amounts of plastics entering the environment. This perspective lays out recent advances to propel both research and action. In this process, phytoaccumulation, phytostabilization, and phytofiltration can be applied to reduce the concentration of nanoplastics and submicron plastics in terrestrial, aquatic, and atmospheric environments, as well as to prevent the transport of microplastics from sources to sinks. Meanwhile, advocating for a more promising future still requires significant efforts in screening hyperaccumulators, coupling multiple measures, and recycling stabilized plastics from plants. Phytoremediation can be an excellent strategy to alleviate global micro/nanoplastic pollution because of the cost-effectiveness and environmental sustainability of green technologies.

## Highlights

• +Current strategies and technological advances in plastic remediation were reviewed.
• Potential applicability of micro/nanoplastic phytoremediation were elaborated.
• Technological processes for micro/nanoplastic phytoremediation were designed.
• Challenges in advancing plastic phytoremediation techniques were proposed.

## Graphical abstract

**Figure:**
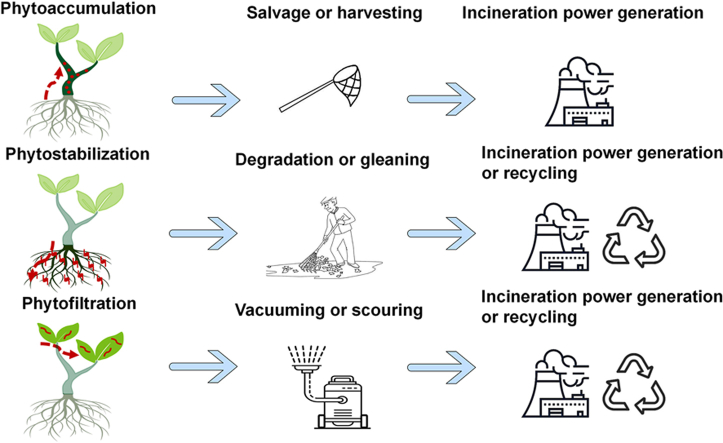

## 1 Introduction

Plastic is one of the hallmarks of the Anthropocene [1]. As of 2015, approximately 6.3 billion tons of plastic waste were generated globally, 79% of which had entered the environment through landfilling, disposal, domestic sewage, and industrial activities [2]. This tremendous amount of plastic pollution is endangering the aquatic, terrestrial, and atmospheric environments, and potentially threatening food safety and human health [3]. Rivers carry 0.8 million to 2.7 million metric tons of plastics from inland to the ocean every year, which is one of the main contributors to marine pollution [4]. Plastics undergo uncontrolled degradation through physical, chemical, and biological processes in the environment, forming microplastics (<5 mm) and nanoplastics (<1 μm) [5]. These plastic particles can carry a panoply of chemical and biological pollutants to various compartments, consequently and potentially threatening ecosystem functioning and services [[6], [7], [8]].

**Fig. 1:**
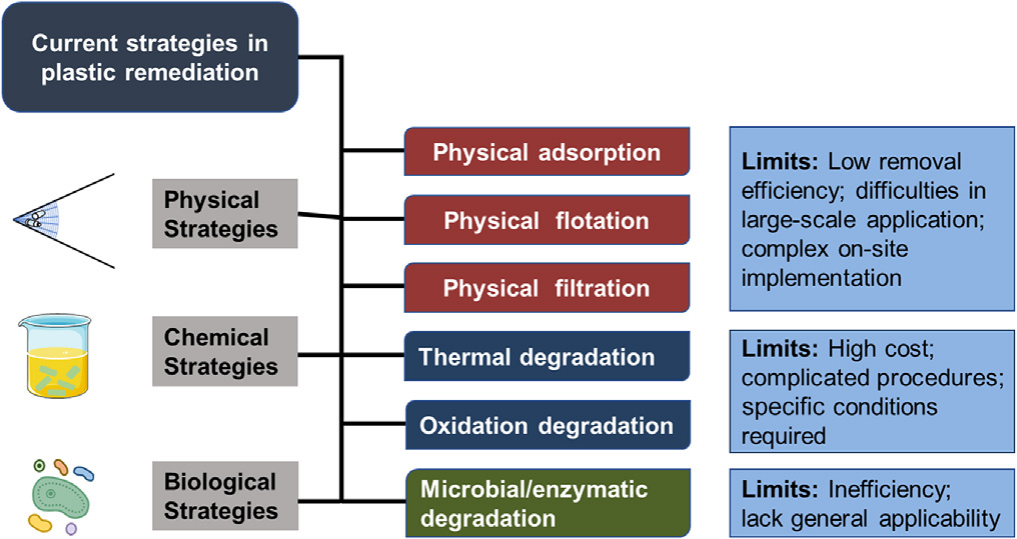
Current strategies and limitations of plastic remediation mainly involve physical, chemical, and biological measures.

Addressing plastic pollution has risen to the forefront of the global environmental agenda [9]. In addition to exploiting alternative products for plastics and degradation approaches for plastic waste, there is an urgent need to intercept and recycle plastic debris in the environment [10]. The current solutions for mitigating plastic pollution in the environment can be identified as physical, chemical, and biological strategies [11]. Among them, physical strategies mainly rely on adsorption, flotation, or filtration to separate plastic wastes from the environment. The chemical and biological strategies advocate for the elimination of recycled plastics through thermal degradation, oxidative degradation, and microbial degradation (Fig. 1). Although many efforts have been made, the existing solutions to alleviate micro/nanoplastic pollution remain incomplete and require future improvement to meet environmental sustainability [12].

Phytoremediation is a cost-effective and environmentally friendly technology that has been successfully applied to the remediation of soil, sediment, and water contaminated with heavy metals or organic pollutants [13]. Recent research on the interaction between plants and plastics has found that plants can intercept and even absorb fine plastics from the contaminated environment [14,15], while also exhibiting high tolerance to long-term exposure to micro/nanoplastics at environmental concentrations [16]. Moreover, other plant-associated effects, such as root exudates and rhizosphere microorganisms, may also provide favorable conditions for the immobilization and degradation of plastics [17,18]. Based on reviewing the recent advances in plastic remediation, this perspective proposes the possible applications and future directions of plastic phytoremediation in terrestrial, aquatic, and atmospheric environments. We hereby call for more future efforts on green technologies for remediating micro/nanoplastic pollution, which have great potential to optimize the existing plastic decontamination frameworks and alleviate the increasing plastic issues while balancing environmental friendliness and sustainability.

## 2 Current strategies and technological advances in plastic remediation

### 2.1 Physical strategies

Separating plastic debris from the environment is the prerequisite for plastic recycling and harmless disposal [19]. Based on the physical characteristics of plastics and site-specific environments, physical remediation techniques such as adsorption, flotation, and filtration could be employed to alleviate plastic pollution in the environment (Fig. 1). The adsorption process has been applied for wastewater treatment [20], but adsorbents oriented toward plastics removal, specifically, are limited. Although microplastics can be adsorbed or immobilized by biochar particles and carbon nanotubes [21,22], this method could introduce new pollutants into the environment. Sun et al. [23] have developed a compressive sponge using chitin and graphene oxide that can adsorb different types of microplastics at a neutral pH. The density-separation method is unsuitable for large-scale field flotation of plastics, while dissolved air flotation (DAF) may be viable for excluding insoluble substances. Conventional DAF has been proven to remove microplastics between 32.0% and 38.0% [24], indicating that the adhesion between microbubbles and microplastics relying on hydrophilic/hydrophobic interactions is not ideal. Disc filtration may be effective at removing larger plastics; however, smaller plastics may still pass through the filter and remain in the treated water [25]. Sand filtration is another practical approach for removing plastic. The sand filter applied by Hidayaturahaman and Lee [26] showed an exclusion efficacy of up to 97% for microplastics. Membrane bioreactor further improved the removal efficiency of microplastics to nearly 99.4% [27]. However, contamination caused by microplastics may bring irreversible membrane fouling [28,29], affecting the sustainability of the implementation of this technology.

### 2.2 Chemical strategies

Advancements in chemical degradation are crucial for the upcycling of plastic waste [30]. Thermal degradation and oxidation degradation are common chemical degradation mechanisms of plastics (Fig. 1). The thermal degradation of plastics is the process of decomposing polymers into oligomers and monomers through the absorption of heat [31]. The thermal degradation of plastic usually occurs under controlled conditions, requiring high temperatures or prolonged heating [30]. Advanced oxidation processes (AOPs), including photochemical oxidation and electrochemical oxidation, are considered promising techniques for eliminating plastic pollution [32]. Photochemical oxidation of plastic requires exposing the polymer to sunlight containing sufficient ultraviolet radiation to trigger the photodegradation of plastics [33]. Notably, one crucial factor in the photodegradation process is the environmental temperature, as it plays a significant role in overcoming the energy barrier of polymers and inducing thermal degradation through the process of thermal oxidation [34]. The main principle of electrochemical oxidation is that the polymer near the anode is oxidized by reactive oxygen species generated by anodic oxidation of water or other reagents (e.g., H_2_O_2_, sulfate, persulfate, etc.) [35,36]. AOP is currently considered a promising solution for plastic pollution control, but the progress is limited due to catalyst constraints.

### 2.3 Biological strategies

Existing biological mitigation strategies for plastics mainly focus on microbial or enzymatic degradation [37]. Many fungi and bacterial strains, such as *Bacillus*, *Rhodococcus*, *Zalerion maritimum*, *and Aspergillus flavus*, have been found to metabolize polymers into monomers [[38], [39], [40]]. Initially, the microbes attach to the surface of plastics and form a plastisphere biofilm [41]. The extracellular enzymes secreted by the microbes (e.g., laccases, peroxidases, lipases, esterase, cutinases, and proteases) then break the chemical bonds of polymers (e.g., C–C, C–N, and –COOR) through enzymatic oxidation and hydrolysis processes [42,43]. Due to their large size, extracellular enzymes can only act on the plastisphere and are unable to penetrate into the plastic. As a result, biodegradation of plastics primarily occurs through pitting [44], which typically initiates within a few days to weeks. For instance, after 28 days of incubation with the fungus *A. flavus* isolated from the guts of the wax moth, about 3.9% weight loss of high-density polyethylene plastics was observed [40]. The biodegradation of plastics is highly influenced by environmental conditions, including pH, temperature, and humidity, as the reproduction and metabolism of microorganisms require the provision of appropriate conditions [38,45]. Moreover, the lack of universality and low efficiency in plastic degradation may limit the application of biodegradation on a large scale in industries.

## 3 Applicability of plants in micro/nanoplastic remediation

### 3.1 Phytoaccumulation of micro/nanoplastics

**Fig. 2:**
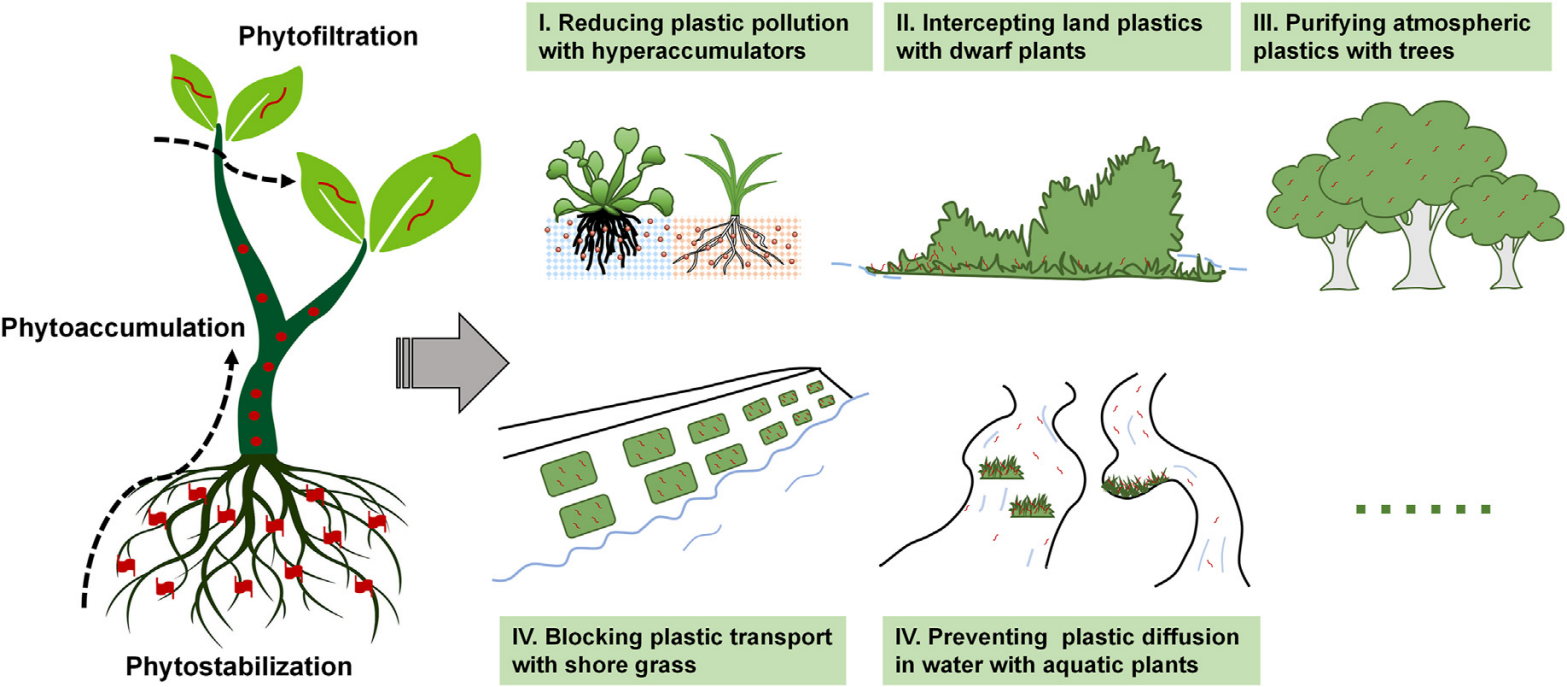
Illustrative examples of the phytoremediation of plastics. Plants may reduce the concentration and/or transportation of micro/nanoplastics in terrestrial, aquatic, and atmospheric environments by phytoaccumulation, phytostabilization, and phytofiltration.

Phytoaccumulation is a method in which plants absorb and accumulate pollutants, thereby reducing the content of pollutants in the environment [46]. The uptake and accumulation of small-sized plastics have been tested in different plants. The root of *Vicia faba* was able to accumulate 100-nm plastic beads with an exposure dose of 100 mg/L after 2 days [47]. Nanoplastics have been shown to accumulate in the cell walls of *Murraya exotica* [48], and have even been observed in the cytoplasm and vacuoles of root cells [49]. Luo et al. [15] quantified the accumulation of submicron plastics (0.2 and 2 μm) in crop plants using europium-doped polystyrene and found that the bioconcentration factors reached 1.8 and 2.9 for wheat and lettuce, respectively. Yuan et al. [16] showed that floating plants like *Eichhornia crassipes* are promising for immobilizing and removing plastics from the water due to their excellent adsorption capability (6,250 μg/g) and high tolerance to plastic exposure. Moreover, recent studies have confirmed the transport process of nanoplastics and submicron plastics in plants. For instance, 100-nm polystyrene particles were observed to be transported from the roots to the shoots and leaves of *Triticum aestivum* [50]. Submicron polystyrene and polymethyl methacrylate particles penetrated the root column through the crack-entry mode, and were transported from the root to shoot driven by the transpirational pull [28,29]. The demonstrated ability of plants to accumulate and transport plastics suggests the viability of employing plants for the removal of nanoplastics and submicron plastics from contaminated environments (Fig. 2).

### 3.2 Phytostabilization of micro/nanoplastics

Plant stabilization is the process of pollutants being adsorbed on the roots or precipitated in the root zone, and being immobilized at the contaminated sites [46]. Although nanoplastics can be directly absorbed by plant roots, microplastics, due to their larger size, are less likely to penetrate plant tissues [51]. Chen et al. [52] reported that plant roots in constructed wetlands could facilitate the interception of microplastics in water flow. The adhesion of microplastics on the surface of macrophytes has been ascertained. For example, 150-μm polyethylene plastics rapidly adhered to a floating plant *Lemna minor*, with an adhesion percentage of microplastics reaching 20% of the total particles [53]. Microplastics were found attached to seaweed and macroalgae in the intertidal zone [54], which may prevent the diffusion of plastics from land to sea. The binding of microplastics on aquatic plants may be attributed to the hydrophobic attraction between plastics and aquatic plants, and the anionic polysaccharides secreted by plants may promote the stabilization of plastics on plant surfaces [55]. The capture of micro/nanoplastics by plant roots and surfaces can reduce their mobility, prevent them from reaching and contaminating other locations, as well as minimize their interactions with other organisms (Fig. 2). Subsequently, by means of rhizosphere degradation and regular collection, phytostabilization may emerge as a viable approach for the removal of micro/nanoplastics from the environment.

### 3.3 Phytofiltration of micro/nanoplastics

Phytofiltration is defined here as vegetation intercepting the flow of plastics through filtration, thereby blocking the unbridled diffusion of micro/nanoplastic pollution (Fig. 2). A basin-wide survey of microplastics demonstrated that grasslands and forests could reduce the abundance of microplastics in freshwater ecosystems [56]. Mangrove wetland is recognized as an intertidal ecosystem that acts as a barrier for retaining land-based plastics [57]. Fibers, foams, and films are dominant microplastics detected in mangrove sediments worldwide [58]. Additionally, the deposition of microplastics on plant leaves presents another pathway for phytofiltration, enabling plants to remove airborne plastic particles. Huang et al. [59] found that the trees with large three-dimensional spaces did have the ability to intercept high-density plastics in the atmosphere. Trees with high coverage (88%) can intercept microfibers and microplastics with an interception rate of approximately 16.3%. Plastic adhesion to plant leaves may be attributed to the electrostatic interaction between plastics and plant biomass. The findings of these studies indicate that phytofiltration holds promise as an *in-situ* remediation approach for the decontamination of plastics in terrestrial, aquatic, and atmospheric systems.

## 4 Opportunities and challenges in advancing phytoremediation techniques

Global plastic pollution has received extensive attention, which in turn initiated important dialogue and spurred innovation toward remedial action to solve the pollution problem. Diverse solutions have been proposed, including a circular economy [60], source reduction via waste management infrastructure [61], and large-scale ocean cleanup [62]. Despite this, even with the implementation of cutting-edge technologies, a tremendous amount of plastic is continuing to enter the environment [63]. To combat ocean plastic pollution, various devices incorporating booms, receptacles, and aquatic vehicles have been developed for trash collection [64]. Nevertheless, implementing such a scheme would entail substantial environmental and economic implications.

Phytoremediation provides an environmentally friendly and low-cost micro/nanoplastic remediation idea, which can greatly supplement the shortcomings of the existing plastic clean-up framework. However, many challenges remain to be addressed as this approach is only starting to be realized. A major problem is the lack of confirmed hyperaccumulators targeting micro/nanoplastics. Recent studies suggested that floating plants, such as *E. crassipes* and *L. minor*, may be potential hyperaccumulators [16,65]. One possible speculation is that developed root systems and vigorous transpiration are beneficial for the uptake of micro/nanoplastics by plants [16]. Screening plant species with phytoremediation potential for micro/nanoplastic decontamination and exploring the uptake and immobilization mechanisms are needed. Current methods for extracting and quantifying micro/nanoplastics in plants are cumbersome and complex. Developing convenient and efficient methods to analyze micro/nanoplastics quantitatively in plants is of paramount importance in screening hyperaccumulation plants and better understanding the remediation process.

The negative response of plants to micro/nanoplastics-induced stress will affect the efficiency and sustainability of the phytoremediation process, while the positive response could facilitate the phytoaccumulation and phytostabilization of micro/nanoplastics by plants. In plant rhizosphere, root exudates and rhizosphere microbes participate in plastic degradation and protect plants from toxic effects, which could contribute to the phytoremediation of micro/nanoplastics [17,38]. Therefore, understanding the mechanism of plant adaptation to plastic pollution and screening plants with better plastic tolerance would provide favorable conditions for the phytoremediation of micro/nanoplastic pollution. Given that plants are fundamental components of ecosystems, it is crucial to acknowledge that consumers may face potential toxic effects when organisms ingest plants that have accumulated micro/nanoplastics. Microplastic has been proven to be a carrier for pollutants (e.g., metals, organic pollutants, and resistance genes) to get into organisms [8]. Hence, prolonged and field experiments are crucial to exploring the interplay between micro/nanoplastics and other contaminants and their impact on the phytoremediation process.

**Fig. 3:**
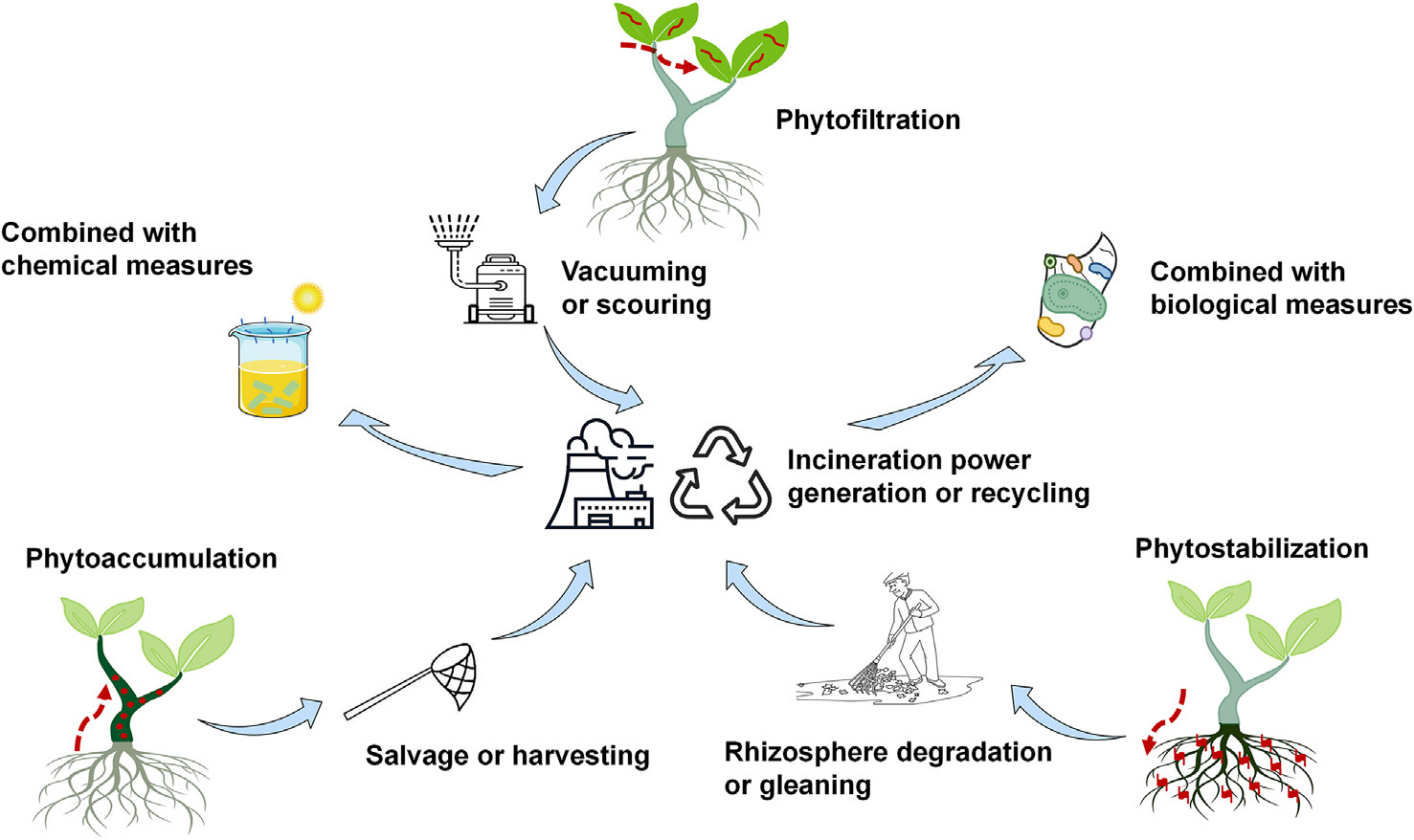
Possible mechanisms and technological process of micro/nanoplastics phytoremediation. The plastics absorbed and intercepted by plants can be recycled through different processes for incineration power generation, reuse, or degradation through other chemical and biological measures.

The advantage of phytoremediation lies in its unique environmental friendliness and sustainability [13]. There are still many aspects that need to be refined to achieve the desired results in phytoremediation. It is important to recognize that phytoremediation serves as a valuable complement to the plastic remediation framework rather than a comprehensive solution on its own. To combat micro/nanoplastic pollution, it is important to address plastic sources, prevent plastic migration, and recycle plastic debris simultaneously. In a realistic environment, plastics of different polymer types and morphology characteristics may exhibit differentiated environmental behaviors. The immobilization of micro/nanoplastic on plant surfaces is not irreversible and may be released again with environmental changes. Therefore, it is necessary to harvest hyperaccumulators and recycle immobilized plastics promptly through means such as salvage, vacuuming, scouring, or gleaning (Fig. 3). To prevent hyperaccumulators from being ingested by organisms and entering the food chain, specific approaches can be to set up quarantine areas, select species outside of animal diets, and collect them in a timely manner. These harvested hyperaccumulators and recycled microplastics can be used for incineration power generation and reuse, thereby reducing micro/nanoplastics pollution in the environment. Additionally, combining phytoremediation with other technologies, such as chemical remediation and microbial degradation, may enhance phytoremediation efficiency and facilitate its wider application and implementation.

## 5 Conclusions

Plastic pollution poses a complex environmental challenge with various land-based sources and few feasible solutions. Scientific understanding and effective solutions have advanced significantly, yet there are still knowledge gaps to address. A transition towards sustainable practices and the “One Health Approach” is likely the key to addressing global plastic pollution. We propose the application of phytoremediation, the power of green technology, utilizing phytoaccumulation, phytostabilization, and phytofiltration, as a potentially promising approach for *in-situ* micro/nanoplastic remediation in terrestrial, aquatic, and atmospheric environments. The priority of phytoremediation strategies will be on elimination technologies for nanoplastics and submicron plastics, as well as recycling technologies for microplastics. Nonetheless, designing and implementing sustainable solutions is a complex task that requires significant future efforts in screening hyperaccumulators, recycling stabilized plastics, and coupling multiple measures.

## CRediT authorship contribution statement

W.K. Y.: conceptualization, writing–original draft, funding acquisition. E.G.B. X.: conceptualization, Writing–review & editing. S. S.: writing–review & editing. P. C.: visualization, software. Y.Y. Y.: resources, supervision, writing–review & editing.

## Declaration of competing interests

The authors declare that they have no known competing financial interests or personal relationships that could have appeared to influence the work reported in this paper.
